# Supplementary material for: Novel Driver Strength Index highlights important cancer genes in TCGA PanCanAtlas patients
Source: PeerJ. 2022 Aug 11;10:e13860. doi: 10.7717/peerj.13860 (PMC9375969; doi:10.7717/peerj.13860)
Supplement: Supplemental Information 2 [file peerj-10-13860-s002.zip › COHORTS/genes plots/age/2021_8_16_14_9_distribution_cohorts_females_65-69.pdf]

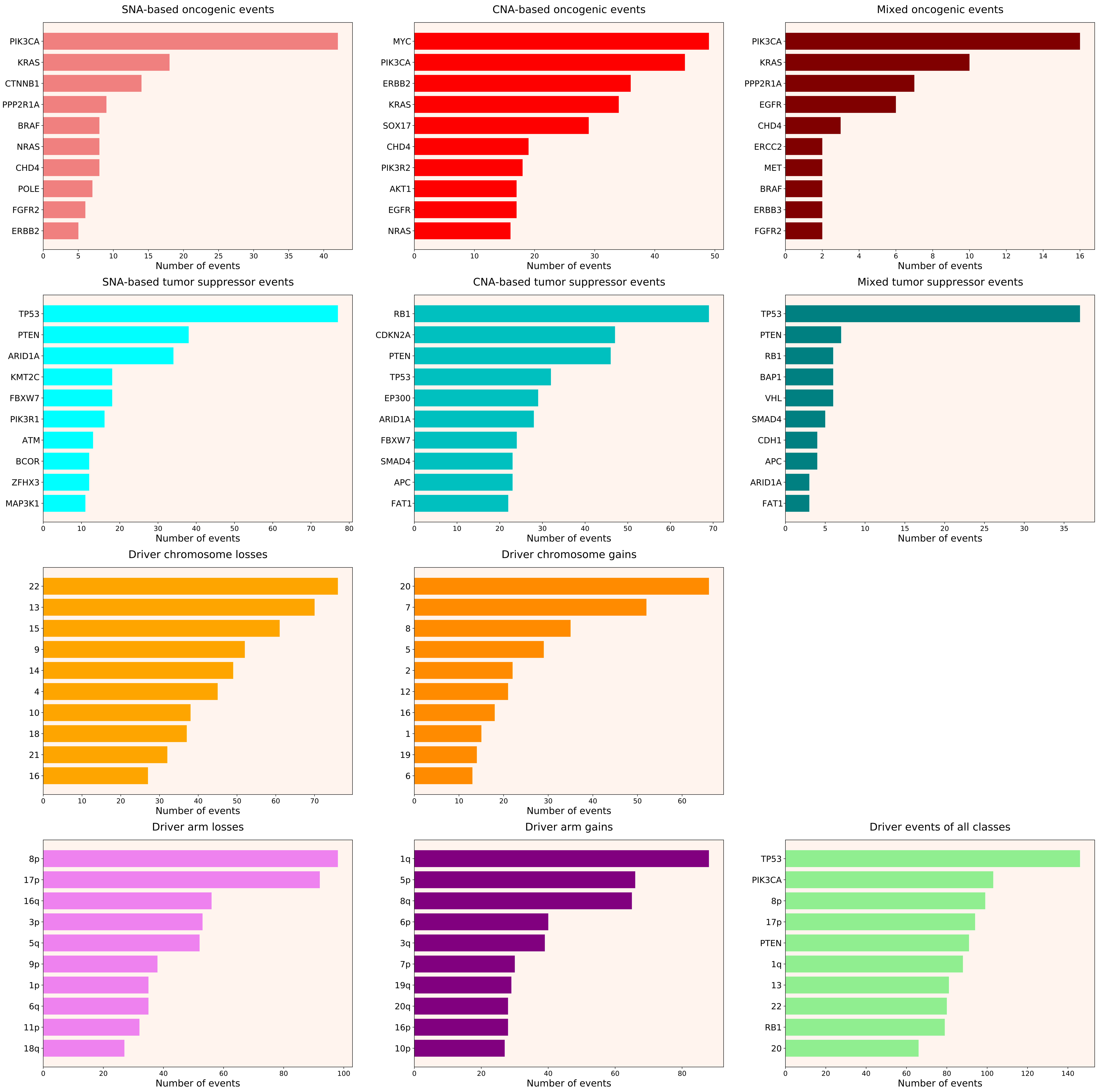
SNA-based oncogenic events
PIK3CA
KRAS
CTNNB1
PPP2R1A
BRAF
NRAS
CHD4
POLE
FGFR2
ERBB2
0 5 10 15 20 25 30 35 40
Number of events
CNA-based oncogenic events
MYC
PIK3CA
ERBB2
KRAS
SOX17
CHD4
PIK3R2
AKT1
EGFR
NRAS
0 10 20 30 40 50
Number of events
Mixed oncogenic events
PIK3CA
KRAS
PPP2R1A
EGFR
CHD4
ERCC2
MET
BRAF
ERBB3
FGFR2
0 2 4 6 8 10 12 14 16
Number of events
SNA-based tumor suppressor events
TP53
PTEN
ARID1A
KMT2C
FBXW7
PIK3R1
ATM
BCOR
ZFHX3
MAP3K1
0 10 20 30 40 50 60 70 80
Number of events
CNA-based tumor suppressor events
RB1
CDKN2A
PTEN
TP53
EP300
ARID1A
FBXW7
SMAD4
APC
FAT1
0 10 20 30 40 50 60 70
Number of events
Mixed tumor suppressor events
TP53
PTEN
RB1
BAP1
VHL
SMAD4
CDH1
APC
ARID1A
FAT1
0 5 10 15 20 25 30 35
Number of events
Driver chromosome losses
22
13
15
9
14
4
10
18
21
16
0 10 20 30 40 50 60 70
Number of events
Driver chromosome gains
20
7
8
5
2
12
16
1
19
6
0 10 20 30 40 50 60
Number of events
Driver arm losses
8p
17p
16q
3p
5q
9p
1p
6q
11p
18q
0 20 40 60 80 100
Number of events
Driver arm gains
1q
5p
8q
6p
3q
7p
19q
20q
16p
10p
0 20 40 60 80
Number of events
Driver events of all classes
TP53
PIK3CA
8p
17p
PTEN
1q
13
22
RB1
20
0 20 40 60 80 100 120 140
Number of events
